# Review of “*Pests and vector-borne diseases in the livestock industry*” edited by Claire Garros, Jérémy Bouyer, Willem Takken and Renate C. Smallegange

**DOI:** 10.1186/s13071-019-3315-0

**Published:** 2019-01-24

**Authors:** Luís Cardoso

**Affiliations:** 1 Department of Veterinary Sciences, School of Agrarian and Veterinary Sciences, UTAD - University of Trás-os-Montes e Alto Douro, Vila Real, Portugal; 2 CECAV - Animal and Veterinary Research Centre, UTAD, Vila Real, Portugal

## Abstract

Claire Garros, Jérémy Bouyer, Willem Takken and Renate C. Smallegange, Editors)

*Pests and vector-borne diseases in the livestock industry*.

Wageningen: Wageningen Academic Publishers; 2018.

611 pages. ISBN 978-90-8686-315-0 (ISSN 1875-0699)

## Review

This book is the 5th edited volume (i.e. a collection of reviewed original scientific articles) within the book series “Ecology and control of vector-borne diseases”. In addition to an introduction (“Livestock pests and vector-borne diseases - a much neglected subject”) and a conclusion (“Control of vector-borne diseases in the livestock industry: new opportunities and challenges”) written by the editors, the collection comprises 18 other chapters organised in four main sections: “Case studies of livestock pests”, “Case studies of vector-borne diseases in livestock”, “State of the art interventions” and “Impact of vector control”. In total, 67 authors (including the four editors) from 18 countries were involved. Thirty-one are from America, 30 from Europe, three from Africa, two from Australia and one from Asia. Eighteen peer reviewers are also listed. The editors explain that “due to a lack of experts available for the review, some topics could not be included (e.g. arthropod pests of swine)”. In fact, it is impossible to cover every single agent in one single book.

In the first section, “Arthropod pests in the poultry industry” presents the main insect and acarine pests in the sector with emphasis on their biology, prevalence, risk to animals and humans, and control methods, including an integrated pest management programme (IPM) and its components (biological and chemical control methods, and education approach for professionals). “Veterinary importance and integrated management of Brachycera flies in dairy farms” considers several species of brachyceran flies, their direct effects and also those as carriers or vectors of pathogens, and measures for control, which further include environmental and mechanical methods. The next chapter, “Acaricides: current status and sustainable alternatives for controlling the cattle tick, *Rhipicephalus microplus,* based on its ecology”, is an extensive review focused on a single tick, stressing the urgent need for sustainable technologies to control infestations and pointing out feasible alternatives to deal with current resistance to acaricides and to promote a safer environment and the quality of animal products. “Sheep myiasis: a one health perspective” summarises causative species and their life-cycles, thereafter relating animal health and welfare with human economic and social aspects.

The second section encompasses another four chapters. “Integrated control of trypanosomosis” comprehensively addresses the question of African animal trypanosomosis integrative control at the community level, which should mainly be based on tsetse control methods, along with diagnosis and medication of vertebrate hosts. “Prevention and control of tick-borne anaplasmosis, cowdriosis and babesiosis in the cattle industry” describes in detail three different tick-borne diseases and the challenges of their control, which must involve a combination of measures. “Mosquito-borne diseases in the livestock industry” gives emphasis to the arboviruses of Rift Valley fever, Japanese encephalitis, West Nile, and the equine encephalitis (western, eastern and Venezuelan), which are all zoonotic. One of the largest chapters in this book, “Case studies of vector-borne diseases in livestock: bluetongue virus”, reviews the control of *Culicoides* spp., which comprises mechanical, chemical, biological, genetic and biotechnological methods.

In the third section, seven additional chapters can be found, starting with “Public-private partnership enabled use of anti-tick vaccine for integrated cattle fever tick eradication in the USA”, which elaborates on anti-tick vaccination of cattle against *R. microplus* and *Rhipicephalus annulatus* with Bm86-based vaccine. “Biological control with parasitoids” approaches biological control with pupal parasitoids as part of an integrated filth fly population management program in conjunction with other control methods in poultry, cattle, equine, swine and other animal facilities. “Biological control of livestock pests: entomopathogens” reviews the status of entomopathogenic fungi, viruses and nematodes for management of pests of livestock and poultry production, including several groups of insects and acarines. “Semiochemical tools for a new generation of livestock pest control” continues the insect and acarine biological control topic, this turn in the form of approaches based on semiochemicals (i.e. behaviour-modifying chemicals) for the surveillance and control of major ectoparasites. “Genetic control of vectors” reviews control methods including the sterile insect technique (SIT), release of insects carrying a dominant lethal gene (RIDL), *Wolbachia*-based and gene drive against insect as pests and pathogen vectors. The implementation of measures to prevent direct contact between livestock and their ectoparasites and minimise associated risks is reviewed in “Biosecurity: methods to reduce contact risks between vectors and livestock”. Closing this section, we can find “The Fly Simulator: a simulation model of stable flies and their control”, which explains the development of an interactive and user-friendly model to better understand the efficient control of stable flies, in the absence or after releases of parasitoids.

The fourth and last main section starts with “Case study: costs of *Culicoides*-borne arboviral diseases” that concentrates on bluetongue, Schmallenberg and African horse sickness, in order to analyse and compare costs of diseases caused by arboviruses transmitted by biting midges of genus *Culicoides*. “Controlling tsetse - what does it cost?” reviews the costs and their calculation methodologies for stationary baits, mobile baits and aerial spraying to control tsetse flies, based on information published since the year 1990. In “Acceptability of vector control actions or co-production of innovations?” the question is analysed in different situations of vector control, namely screw-worms, tsetse, stoxomes and ticks, and with inputs of social psychology and political sciences.

In general, while they have different sizes, all chapters are well written and organised, with a list of comprehensive literature, and most of them are rich in figures and also informative tables. Repetition is not an issue and reading is enjoyable and very instructive, although subheadings could have been numbered to facilitate understanding (especially in chapter 18). Very few typos could be detected (e.g. Ichnocera, p. 38), but genus *Boophilus* should be *Rhipicephalus* in chapter 4 (Table 1) and chapter 12 (p. 340).

The book is mainly aimed at parasitologists, entomologists, microbiologists and epidemiologists, and is a must-have for libraries of teaching and research institutions. Those who already have other volumes should buy this one; those who do not yet have any volume can start the collection from now on.

